# Cloning and Functional Analysis of the Promoter of an *Ascorbate Oxidase* Gene from *Gossypium hirsutum*

**DOI:** 10.1371/journal.pone.0161695

**Published:** 2016-09-06

**Authors:** Shan Xin, Chengcheng Tao, Hongbin Li

**Affiliations:** College of life sciences, Key laboratory of Agrobiotechnology, Shihezi University, Shihezi, Xinjiang, China; USDA-ARS Southern Regional Research Center, UNITED STATES

## Abstract

Apoplastic ascorbate oxidase (AO) plays significant roles in plant cell growth. However, the mechanism of underlying the transcriptional regulation of *AO* in *Gossypium hirsutum* remains unclear. Here, we obtained a 1,920-bp promoter sequence from the *Gossypium hirsutum* ascorbate oxidase (*GhAO1*) gene, and this *GhAO1* promoter included a number of known cis-elements. Promoter activity analysis in overexpressing *pGhAO1*::*GFP-GUS* tobacco (*Nicotiana* *benthamiana*) showed that the *GhAO1* promoter exhibited high activity, driving strong reporter gene expression in tobacco trichomes, leaves and roots. Promoter 5’-deletion analysis demonstrated that truncated *GhAO1* promoters with serial 5’-end deletions had different GUS activities. A 360-bp fragment was sufficient to activate GUS expression. The P-1040 region had less GUS activity than the P-720 region, suggesting that the 320-bp region from nucleotide -720 to -1040 might include a cis-element acting as a silencer. Interestingly, an auxin-responsive cis-acting element (TGA-element) was uncovered in the promoter. To analyze the function of the TGA-element, tobacco leaves transformed with promoters with different 5’ truncations were treated with indole-3-acetic acid (IAA). Tobacco leaves transformed with the promoter regions containing the TGA-element showed significantly increased GUS activity after IAA treatment, implying that the fragment spanning nucleotides -1760 to -1600 (which includes the TGA-element) might be a key component for IAA responsiveness. Analyses of the *AO* promoter region and *AO* expression pattern in *Gossypium arboreum* (Ga, diploid cotton with an AA genome), *Gossypium raimondii* (Gr, diploid cotton with a DD genome) and *Gossypium hirsutum* (Gh, tetraploid cotton with an AADD genome) indicated that *AO* promoter activation and *AO* transcription were detected together only in D genome/sub-genome (Gr and Gh) cotton. Taken together, these results suggest that the 1,920-bp *GhAO1* promoter is a functional sequence with a potential effect on fiber cell development, mediated by TGA-element containing sequences, via the auxin-signaling pathway.

## Introduction

Upland cotton (*Gossypium hirsutum L*.*)* is an important economic crop worldwide and occupies a vital position in the global economy as its fibers are the most important plant materials for the textile industry [1]. Cotton fiber develops from the seed coat as a single epidermal cell, and the process of fiber development can be divided into four overlapping stages: initiation, elongation, secondary wall deposition and maturation [2]. The plant hormone auxin performs a decisive function in fiber development by regulating extracellular oxidative signals that affect cell wall configuration [3–5].

As a member of a small multigene family of multicopper oxidases, ascorbate oxidase (AO; EC 1.10.3.3) catalyzes the oxidation of ascorbic acid (AA) to dehydroascorbate (DHA), thereby generating oxidative signals in apoplasts [6–8]. DHA is an important oxidative molecule in apoplasts, and many studies have suggested that the oxidative signal catalyzed by AO plays a crucial role in cell elongation and enlargement [9]. AO is strongly expressed in the stretch expanded fruit of cucurbitaceous plants, including cucumber, pumpkin and melon. In pumpkin, *AO* expression is promptly increased during callus growth, fruit development and seedling elongation [9,10]. *AO* is also abundantly expressed in the young and growing tissues of tobacco [6,11].

Auxin signal transduction is closely associated with the apoplast redox state, which is modulated by AO. An auxin-binding protein (ABP1) present on the apoplast crosses the plasmalemma and is crucial for auxin-induced responses, and auxin responsiveness can be suppressed when there is an excess of oxidized ascorbate in apoplasts [12,13]. Auxin generates an abundance of oxidative signals through a comprehensive network in which AO might serve indispensable functions [9]. In plants, a flexible redox equilibrium in the apoplast is a key signal for determining plant cell sensing, transducing external environmental changes and activating hormone signal pathways [13]. Oxidative signals include oxygen-containing molecules, such as ROS, and non-oxygen-containing molecules, such as DHA, and both auxin and ROS signals play important roles in the fast elongation development of cotton fibers [3,13,14]; however, the precise mechanism underlying this process remains obscure.

Previously, we reported that cotton ascorbate peroxidase is closely associated with fiber elongation development in response to ROS and ethylene [14]. In the present study, the promoter of an ascorbate oxidase gene, *GhAO1*, was obtained. The promoter effectively drove the expression of *GUS* and *GFP* in tobacco trichomes. Functional sequences within the *GhAO1* promoter were uncovered using serial 5’-end deletion. The *GhAO1* promoter contains an auxin-responsive cis-acting element (TGA-element) and shows induced activity under IAA treatment. We concluded that the *GhAO1* promoter is a functional sequence potentially involved in fiber development via the auxin-signaling pathway.

## Materials and Methods

### Growth of plants and material harvest

Upland cotton (*Gossypium hirsutum* L. cv. Xuzhou 142) seeds, tobacco (*Nicotiana* *benthamiana*) seeds, *Escherichia coli* strain DH5α, pCAMBIA1304 plasmid and *Agrobacterium tumefaciens* strain GV3101 were used in the present study and were maintained at the Key laboratory of Agrobiotechnology of Shihezi University. Cotton and tobacco plants were grown in a greenhouse at 28°C with a natural photoperiod.

### Cloning of *GhAO1* promoter

The cotton genomic DNA used for genome walking was isolated as described method [15]. Genome walking was performed to isolate the *GhAO1* promoter region using a TAIL-PCR method and the Genome Walking kit (TaKaRa, Dalian, China) according to the manufacturer’s instructions. The random primers were improved according to a previous study [16], and the three specific primers (SP1, SP2 and SP3) listed in Table 1 were designed using the *GhAO1* cDNA sequence (GenBank Accession number: KT794559).

**Table 1:** Primers used in the present study.

Primer name	Primer sequence
Isolation of *GhAO1* promoter	
LAD	ACGATGGACTCCAGAGCGGCCGCNNNCGGT
AC1	ACGATGGACTCCAGAG
SP1	GTATTCGAAAGCCTCTCCTGGGT
SP2	CAGTGAATGACAACTCCCTCGGT
SP3	CTCCGTACGTGTATTCCACTTCC
Vector construction*	
P-1920-Foward	CGGGATCCTGTGCTTTATCAACTCATTGA
P-1600-Foward	CGGGATCCCAACTCATGAA GTTAAAAAAA
P-1320-Foward	CGGGATCCGCACCTCCATTACTAATTATT
P-1040-Foward	CGGGATCCTAAAATTAATTGATCTGATTT
P-720-Foward	CGGGATCCATCTATTACCACAATTTTACA
P-360-Foward	CGGGATCCATCTAATCCCAGTTCATTTGT
FP-Reverse	GAAGATCTGTTTCAGTACGTAAAACCAGG
RT-PCR	
GaAO-Foward	GAGTCAGTGAGCGAGGAAGCG
GaAO-Reverse	CCCTGGAACCCCAAGATTTA
GrAO-Foward	ATGGGGATGGGGGTTATTTT
GrAO-Reverse	CAGTCTCTCAGTTCTGCTTGTTTC
UBQ7-Foward	GAAGGCATTCCACCTGACCAAC
UBQ7-Reverse	CTTGACCTTCTTCTTCTTGTGCTTG

* Restriction sites of *Bam*H I in forward primers and *Bgl* II in reverse primers were underlined.

The extracted genomic DNA was subjected to pre-amplification using the primers LAD and SP1, and the product was subsequently diluted and used as a template in primary TAIL-PCR using the primer pairs AC1 and SP2. After secondary TAIL-PCR using the diluted primary TAIL-PCR product as a template and the primers AC1 and SP3, the amplified products were analyzed on 1.0% agarose gels, and single fragments were isolated from the gels and purified using a DNA purification kit. A ~2000-bp fragment was ligated to pMD19-T vector and transformed into *E*. *coli* for sequencing. The PLANTCARE database (http://bioinformatics.psb.ugent.be/webtools/plantcare/html/) was used to identify potential cis-regulatory elements within the promoter.

### Construction of the expression vector

A 1,920 bp fragment (pGhAO1) upstream of the translational start codon of the *GhAO1* gene and 5'-truncated promoter sequences were obtained by PCR using a single reverse primer and different forward primers (listed in Table 1) carrying *Bam*H I and a *Bgl* II restriction sites. The amplified fragments were inserted upstream of the *GUS* gene coding region of the plasmid pCAMBIA 1304 (Clontech) as *Bam*H I-*Bgl* II fragments at the corresponding restriction sites, replacing the cauliflower mosaic virus (CaMV) 35S promoter, producing a series of *pGhAO1*::*GFP-GUS* vectors, namely P-1920 (-1920/-1, 1920 bp), P-1760 (–1760/-1, 1760 bp), P-1600 (-1600/-1, 1600 bp), P-1320 (-1320/-1, 1320 bp), P-1040 (-1040/-1, 1040 bp), P-720(-720/-1, 720 bp), and P-360(-360/-1, 360 bp). After verification by sequence analysis, the confirmed constructs were used to determine the promoter activity in transgenic tobacco plants.

### Transformation of tobacco plants

The eukaryotic expression vector *pGhAO1*::*GFP-GUS* (P1920) was introduced into *A*. *tumefaciens* GV3101 using a freeze-thaw method. Transgenic tobacco plants were generated using an agrobacterium-mediated leaf disk transformation regeneration method [17]. The transformed tobacco plants were selected on MS medium supplemented with 250 mg/L of carbenicillin disodium and 30 mg/L of hygromycin, and hormone-free MS medium containing 250 mg/L of carbenicillin disodium was used for plant regeneration. The identified transgenic plants were transferred to soil for blossom growth and seed bearing. All plants were grown in the greenhouse at 26°C with a 16 h light/8 h dark cycle. The constructed vectors were transformed into tobacco leaves using the transient transformation method [18], and the generated transgenic leaves were utilized for GUS activity analyses.

### Histochemical GUS staining and GUS activity quantification

Histochemical localization and GUS enzyme activity were determined as previously described [19]. Plant tissues, including the leaves and roots of 6–7 week-old transgenic tobacco seedlings, were immersed in GUS staining buffer with successive incubation overnight at 37°C and de-staining in ethanol. X-gluc was dissolved in dimethyl sulfoxide (DMSO) and diluted 20-fold in substrate solution. The images were obtained using a microscope (Olympus,Japan). Fluorometric analysis of GUS activity was performed using 4-methylumbelliferyl-b-glucuronide (4-MUG) as a substrate. The extracted proteins from transgenic leaf samples were mixed with GUS assay buffer (2 mM 4-MUG, 50 mM sodium phosphate buffer pH 7.0, 10 mM β-mercaptoethanol, 10 mM Na_2_EDTA, 0.1% sodium lauroyl sarcosine, and 0.1% Triton X-100). The protein concentration was determined using the Bradford protein assay. The reaction was held at 37°C and terminated upon the addition of stop buffer (0.2 M Na_2_CO_3_). GUS activity was determined in triplicate based on the detection of 4-methylumbelliferone fluorochrome (4-MU) generated by the GUS-mediated catalysis of 4-MUG hydrolysis using a fluorescence spectrophotometer with an excitation wavelength of 365 nm and an emission wavelength of 455 nm. The GUS activity was defined as pmol of 4-methylumbelliferone per mg protein per min.

### GFP localization

The leaves of 6–7 week-old transgenic tobacco plants were selected for the analysis of GFP localization. A confocal laser-scanning microscope (Zeiss LSM510, Germany) was used to monitor GFP localization. A laser at the wavelength of 488 nm was used to activate GFP fluorescence.

### IAA treatment

The transgenic tobacco leaves transformed using a transient expression method were grown in an artificial climate incubator for 6–7 weeks on MS medium, and subsequently placed under a white fluorescent lamp for 1 h with successive inoculation at 25°C in MS medium with or without 1 mg/L IAA treatment for 2 days; the resultant materials were used for further GUS activity analysis.

## Results

### Sequence analysis of the *GhAO1* promoter

**Fig 1:**
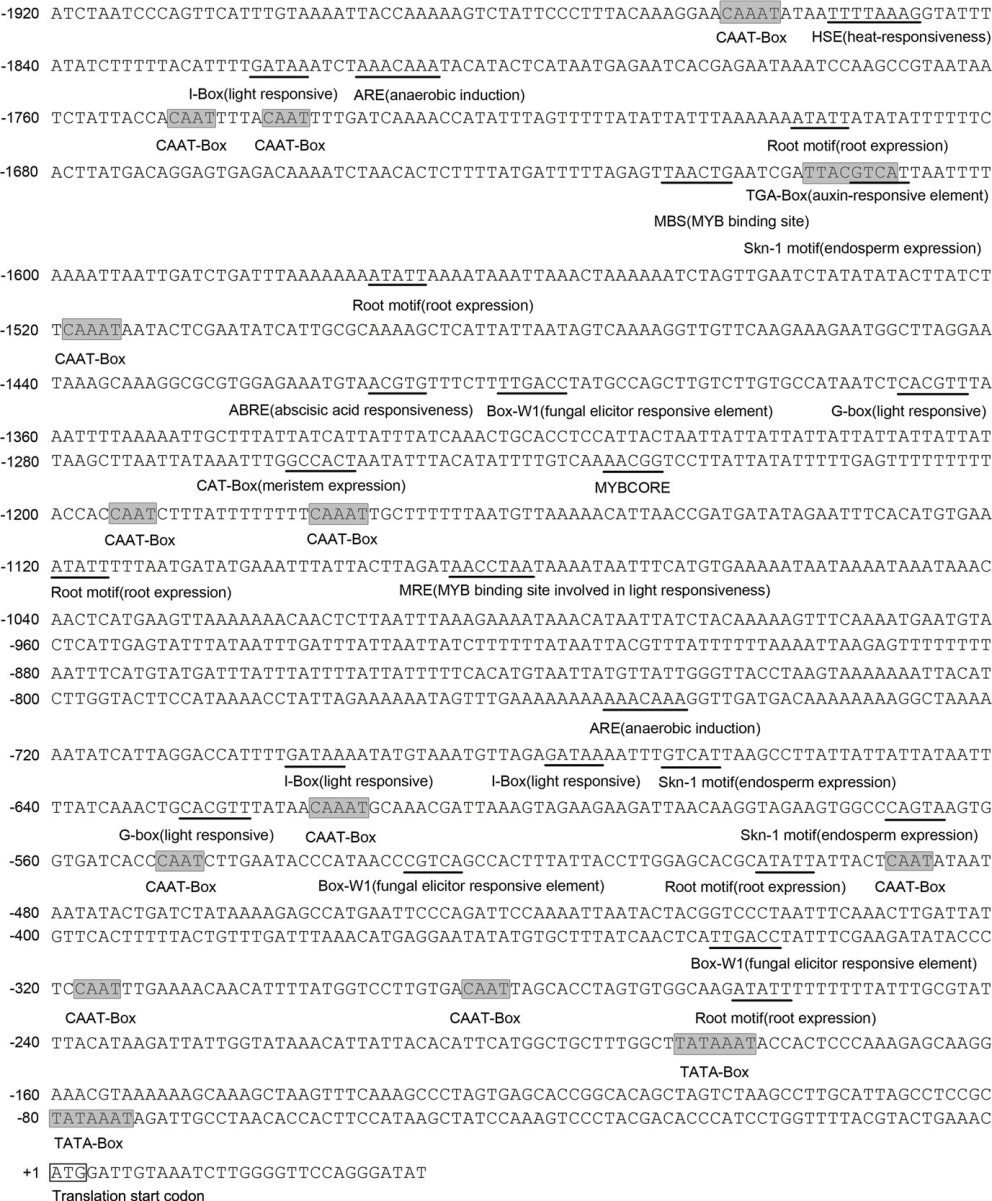
Analysis of the *GhAO1* promoter sequence. The translation start codon is indicated by a box. The putative TATA-box and CAAT-box are indicated by shading, and the CAT-box, Skn-1 motifs, root motifs, MYB recognition sites, G-box, ARE, TGA-element, ABRE and HSE are indicated by underlining.

We obtained an ascorbate oxidase gene *GhAO1* (GenBank accession number: KT794559) from fast elongating fiber tissues previously. *GhAO1* was specifically accumulated during fiber fast elongation development stages (5–20 dpa) in widetype (WT) cotton ovules associated with fibers compared with 10-dpa *fuzzless-lintless (fl)* mutant ovules, both at mRNA level and enzyme activity (S1 Fig). With reference to the cDNA sequence of *GhAO1*, a 1,920-bp sequence upstream of the coding region was isolated using the genome walking method. Sequence analysis using the online program PLACE (http://dna.affrc.go.jp/PLACE) revealed that a number of putative plant cis-elements were present. As shown in Fig 1, two TATA-boxes were identified, at the -74 and -181 bp sites, and seven CAAT-boxes were identified, at the -282, -315, -486, -548, -1,192, -1,739 and -1,747 bp positions. Some hormone-related elements were also recognized, including the auxin-responsive element (TGA-element: AACGAC) and abscisic acid responsive regulatory motif (ABRE: CGTACGTGCA). Light-responsive elements, such as the I-box (ATGATATGA), MRE (MYB binding site: AACCTAA) and G-box (CACGTT), were also observed. For tissue- or developmental stage-specific motifs, two Skn-1 motifs (endosperm expression: GTCAT), one CAT-box motif (meristem expression: GCCACT) and three root motifs (root expression: ATATT) were explored. Heat responsive elements (HSEs) (TTTTAAA), MYB recognition sites containing an MBS (MYB binding sites) (TAACTG) and MYBCORE elements (AACGG) were also identified in the present study.

### *GhAO1* promoter activity analysis

**Fig 2:**
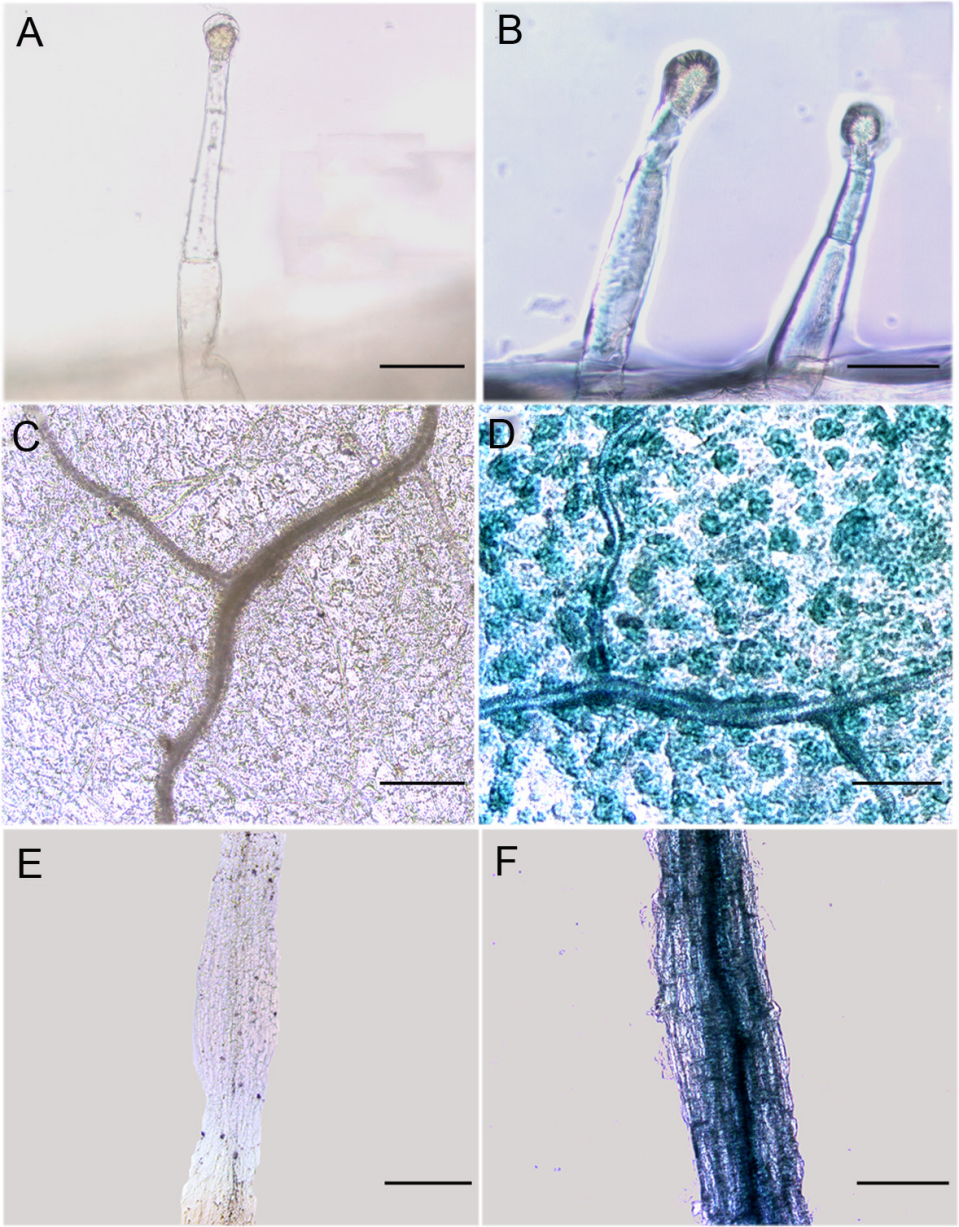
Histochemical staining of GUS in transgenic tobacco plants expressing *pGhAO1*::*GFP-GUS*. (A) Hair from the leaf epidermis of non-transgenic tobacco plants. (B) Hair from the leaf epidermis of transgenic tobacco plants. (C) Representative leaf from non-transgenic tobacco plants; (D) Representative leaf from transgenic tobacco plants. (E) Representative root from non-transgenic tobacco plants. (F) Representative root from transgenic tobacco plants. Bars = 10 μm (in A, B), 50 μm (in C, D) and 100 μm (in E, F).

**Fig 3:**
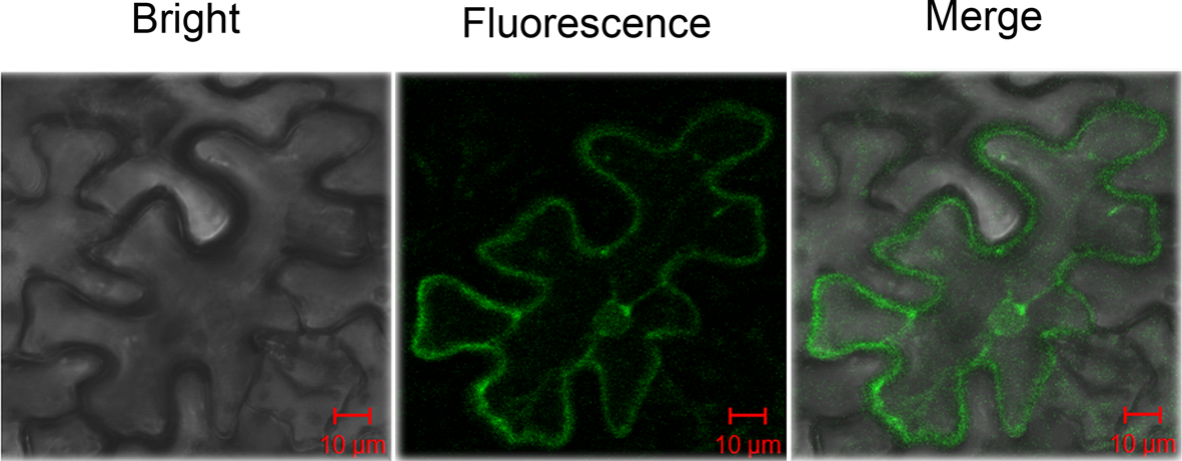
Fluorescence detection of GFP expression by transgenic tobacco leaves. The leaves of *pGhAO1*::*GFP-GUS* transgenic tobacco plants were used to determine GFP expression (bright field, fluorescence and merged images). The GFP signals were detected by fluorescence microscopy.

To evaluate the activity of the *GhAO1* promoter, *pGhAO1*::*GFP-GUS* was constructed and transformed into tobacco by an agrobacterium-mediated leaf disk method, and materials of identified transgenic tobacco plants were used for activity analysis. The activity of the integrated *GhAO1* promoter, detected by histochemical GUS staining, showed that leaf epidermal hairs (Fig 2A and 2B), leaves (Fig 2C and 2D) and roots (Fig 2E and 2F) turned blue in transformed tobacco plants, indicating that the *GhAO1* promoter markedly drives GUS expression. The green fluorescence of transformed tobacco leaves was detected using a fluorescence microscope (Fig 3), revealing that the *GhAO1* promoter effectively activates reporter gene *GFP* expression in tobacco. The results demonstrated that the *GhAO1* promoter is an integral functional sequence.

### Functional analysis of the regulatory regions of the *GhAO1* promoter

**Fig 4:**
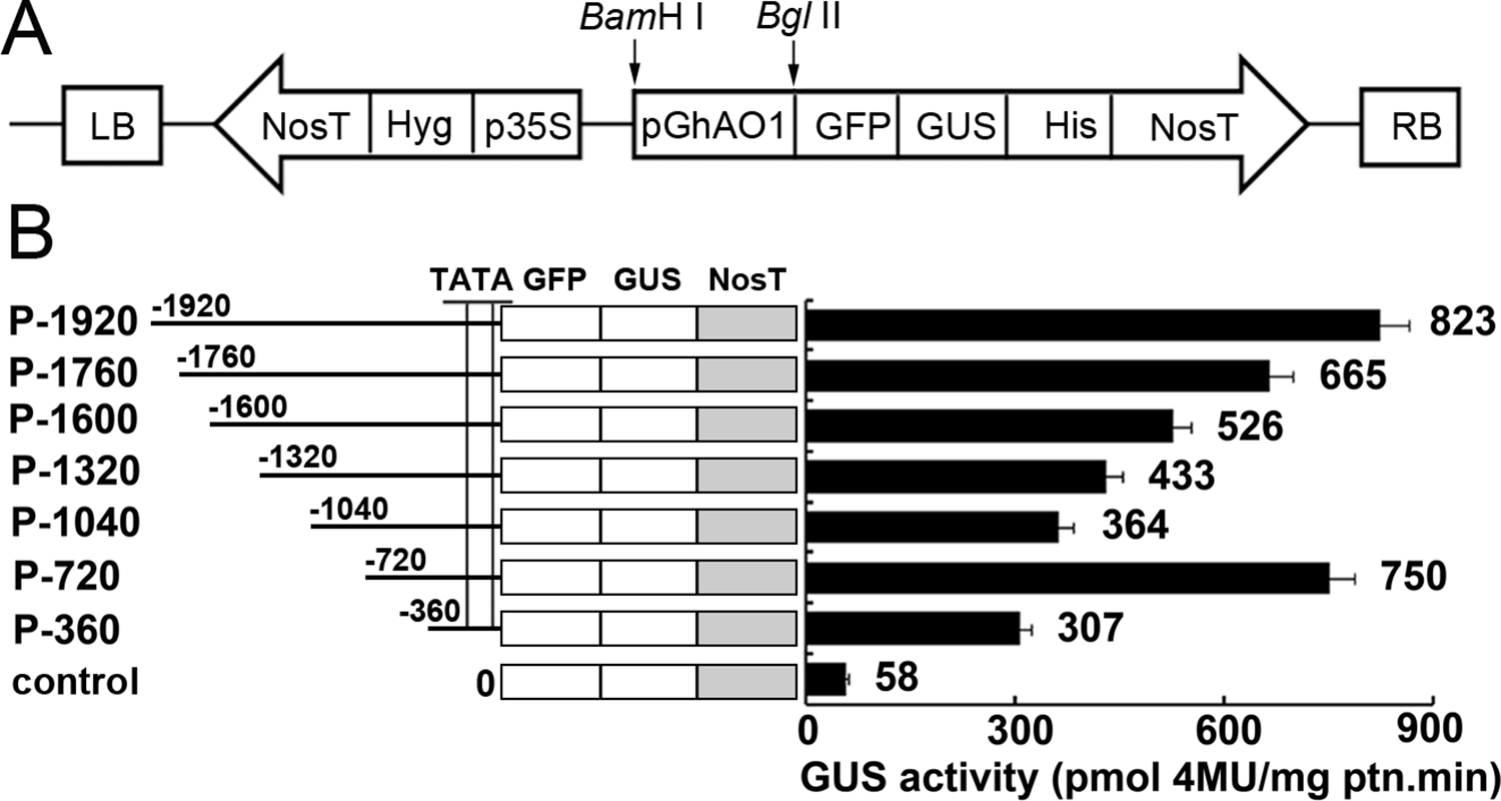
Promoter activity analysis of the *GhAO1* promoter and 5’-deletion constructs. Different constructs were transformed into tobacco, and GUS activity was assayed. (A) Schematic presentation of the 5’-deletion constructs. The full-length and truncated fragments were fused to the *GFP-GUS* gene, generating the constructs P-1920, P-1760, P-1600, P-1320, P-1040, P-720, and P-360. (B) Quantitative analysis of the GUS activity of the constructs. The promoter activity was determined in transgenic tobacco leaves transformed with the different constructs. The specific GUS activity was determined as the rate of 4-methylumbelliferyl β-D-glucuronide conversion to 4-methylumbelliferone (pmol mg protein^-1^ min^-1^). The data are presented as the average of three independent experiments.

To determine the activity of the various regulatory regions of the *GhAO1* promoter, a series of 5’-deletion constructs of the promoter region were fused with the *GUS* gene to obtain *GhAO1* promoter-GUS chimeric genes, in accordance with the schematic shown in Fig 4A. The promoter activities of P-360, P-720, P-1040, P-1320, P-1600, P-1760, and P-1920 were significantly higher than that of the control (Fig 4B). The GUS activity of P-720 was approximately 2-fold higher than that of P-1040, indicating that the 320-bp region from nucleotides -720 to -1040 bp might include at least one cis-element that acts as a silencer.

### IAA response analysis of the *GhAO1* promoter

**Fig 5:**
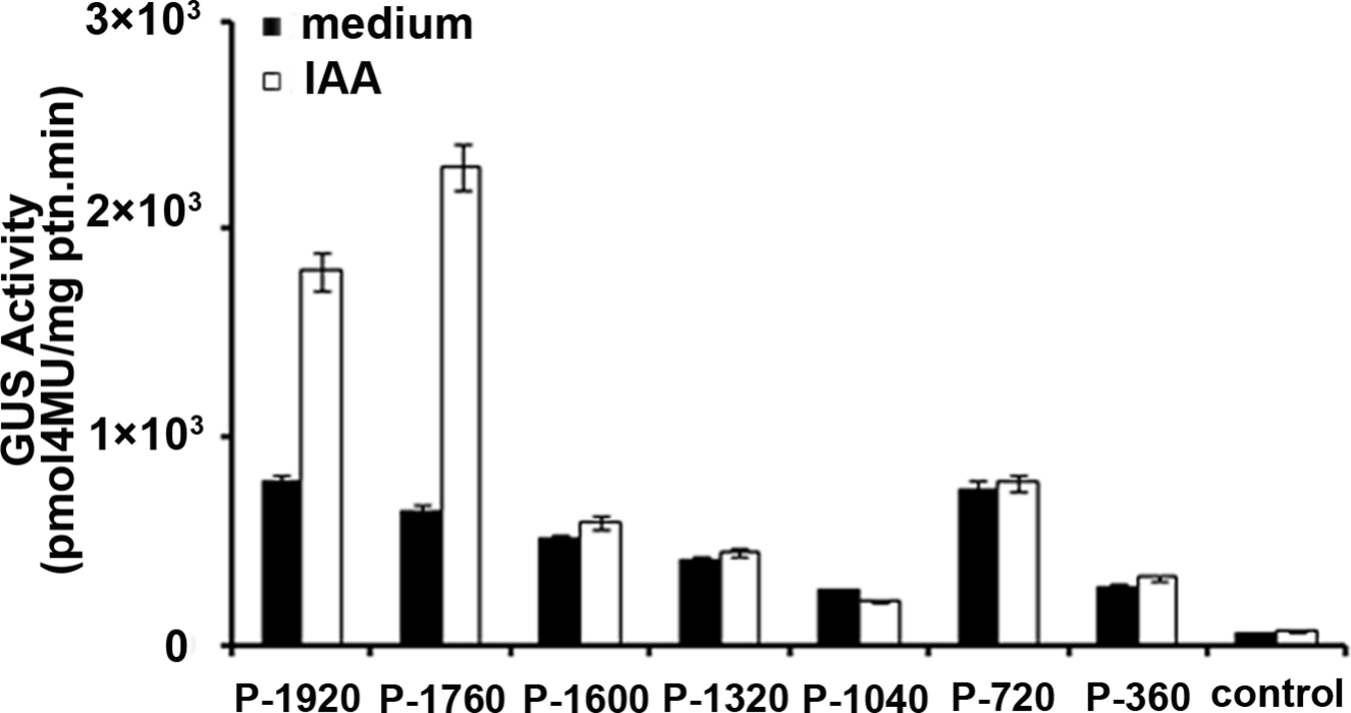
Promoter activity analysis of serial 5’-deletion constructs of the *GhAO1* promoter under IAA treatment. Various 5’-deletion constructs were transformed into tobacco leaf discs (1.5 cm diameter) using the agrobacterium-mediated transient transformation method. Discs of tobacco leaf tissue were incubated in MS medium with or without 1 mg/L IAA and incubated at 25°C for 2 d. Then, the quantitative analysis of GUS activity was spectrophotometrically measured. The GUS activity of non-DNA transformants (control) was also investigated. The white columns show GUS activity under normal treatment, and the black columns show GUS activity under IAA treatment. Each value represents the mean of the results from three independent experiments, and the bars indicate standard deviations.

Previous studies have demonstrated that transcription of the *AO* gene is induced by auxin. The *GhAO1* promoter contains an auxin-responsive cis-acting element (TGA-element). To confirm the relationship between the regulatory region of the cotton *GhAO1* promoter and auxin, a series of 5’-end deletion constructs designed to contain or not contain the auxin-responsive cis-acting element were transformed into tobacco leaf tissues, and the obtained transgenic tobacco leaves were incubated in Murashige-Skoog (MS) medium with or without 1 mg/L IAA at 25°C for 2 days. The GUS activity of these transformed leaf tissues was measured using non-DNA transformants as controls. In control leaf tissues, almost no GUS activity was detected, whereas higher GUS activity was measured in all transformed leaf tissues, regardless of IAA treatment. There were no obvious differences in the GUS activity in the P-360, P-720, P-1040, P-1320 and P-1600 transformants, which did not contain the auxin-responsive cis-acting element, under water or IAA treatment. Interestingly, in the P-1920 and P-1760 transformants, containing the auxin-responsive cis-acting element, the GUS activity in leaf tissue incubated in the presence of 1 mg/L IAA was approximately 3- to 4-fold higher than in the absence of 1 mg/L IAA. Furthermore, this GUS activity was approximately 6-fold higher than that in the non-DNA transformed control (Fig 5). However, the increased GUS activity induced by IAA disappeared in the P-1600 transformant and shorter construct transformants, indicating that the fragment from -1760 to -1600 bp, containing the cis-acting auxin responsive element, is functional.

### Sequence analysis of *AO* promoter and expression pattern of *AO* gene in *Gossypium arboreum* (Ga), *Gossypium raimondii* (Gr), and *Gossypium hirsutum* (Gh)

**Fig 6:**
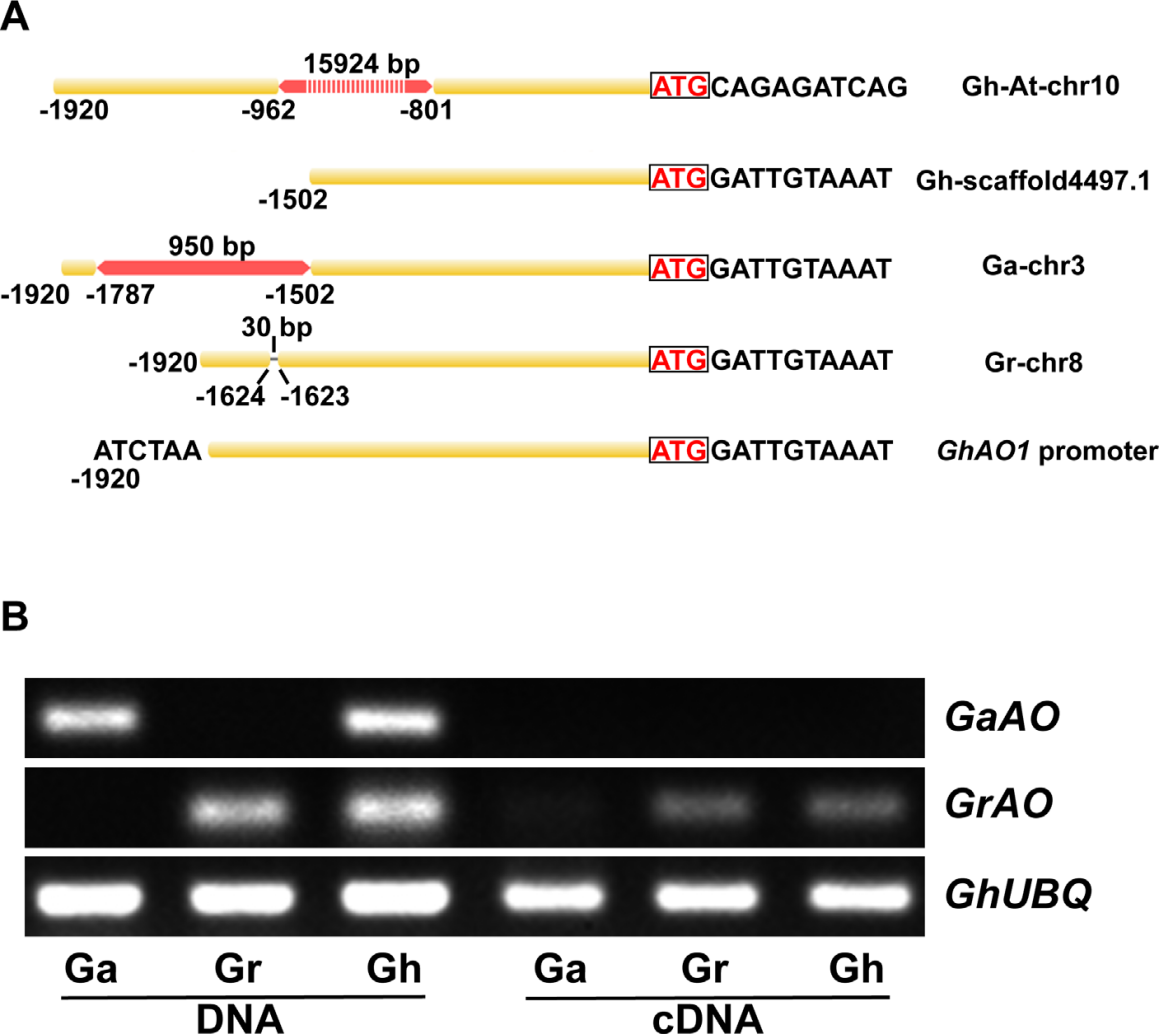
Comparison of the *GhAO1* promoter region and *GhAO1* expression patterns in Ga, Gr and Gh cotton. (A) Sequence structure of the *GhAO1* promoter regions in the At, Dt, A and D genomes. A 30-bp gap was detected in Gr (D genome) at -1623 bp. A 950 bp fragment replaced the 284 bp (from -1502 to -1787 bp) region of the *GhAO1* promoter in Ga (A genome). In the tetraploid cotton Gh, two copies of the *GhAO1* promoter were detected in the At and Dt genomes. The 418-bp of the 5’ region of the *GhAO1* promoter is missing in the Dt sub-genome, while a 15924 bp gap replaces the 160 bp (from -801 to 962 bp) region of the *GhAO1* promoter. Yellow, homologous regions; red, substituted sequences; dashed line, gaps. The start codons are framed and labeled in red. (B) The expression patterns of *GaAO and GrAO* transcripts from the DNA and cDNA templates of Ga, Gr and Gh cotton plants. The primers were specifically designed for *GaAO* amplification from Ga and Gh DNA templates, while the primers specifically designed for *GrAO* were designed for PCR amplification from DNA and cDNA templates. *GhUBQ7* was used as a reference gene. Template materials were extracted from 10-d post-anthesis fibers. The primers used in the present study are listed in Table 1.

Sequence analysis and RT-PCR were performed to investigate the linear structure of the *AO* promoter and the expression pattern of the *AO* gene in Ga, Gr and Gh. The 1,920-bp *GhAO1* promoter matched the 5485295–5487167 genome region of chromosome 8 in Gr (almost 100% identity, with a 30-bp gap at -1623 bp); the 89397689–89400248 genome region of chromosome 3 in Ga (with a 950-bp region, instead of the 285-bp region from -1502 to -1787); the 23773–25242 genome region of scaffold 4497.1 (missing -1503 to -1920, a potential component of the Dt sub-genome) in Gh; and the 76757247–76773171 genome region of At chromosome 10 (with a 15924-bp gap, instead of the 161-bp region from -801 to -962 bp of the *GhAO1* promoter) in Gh (Fig 6A). Gaps were detected in both the A genome (Ga) and At sub-genome (Gh), while high identities were observed in the D genome (Gr) and Dt sub-genome (Gh), indicating that the *GhAO1* gene from the D or Dt genome was activated. The 950 and 15924-bp gaps might dramatically reduce the expression of the *AO* gene from the A or At genome in both Ga and Gh, according to the functional analysis of the regulatory region of the *GhAO1* promoter (Fig 4). Furthermore, the expression patterns of the *AO* gene from the D and A genomes were examined using RT-PCR. Primers specific for the *AO* sequences from genomes A and D were used to amplify both DNA and cDNA templates. The PCR products obtained using the *GaAO*-specific primers were detected only with the Ga and Gh DNA templates, while no bands were observed for any of the cDNA templates (Fig 6B). For the *GrAO* primers, the products were not only amplified from DNA templates but also from cDNA templates (both Gr and Gh), suggesting that the *GhAO1* promoter from the D genome/sub-genome was activated (Fig 6B). Our results indicate that the sequence containing the auxin responsive element might be a key regulator of promoter-driven expression and transcriptional regulation of the *GhAO1* gene.

## Discussion

Ascorbate oxidase plays important roles in redox state maintenance and oxidative burst generation in apoplasts, thereby controlling cell division and expansion. *AO* is highly expressed in fast-growing organs, indicating a direct link between *AO* and cell development through regulating redox balance regulation of the apoplast [18]. However, the mechanisms for the regulation of apoplastic *AO* expression and the promotion of cell growth remain unclear. In the present study, we isolated the promoter of the cotton *GhAO1* gene to investigate the regulation of *GhAO1*. Sequence analysis showed that the promoter region contains some typical plant cis-elements important for *GhAO1* transcriptional regulation (Fig 1); these might affect cell growth by modulating *GhAO1* gene expression.

*AO* cDNAs have been isolated from many plants, such as cucumber, pumpkin, tobacco, and melon [11,20–23]. Although the consequences of cell elongation in *AO* overexpressing tobacco plants (via increased AO enzyme activity and DHA concentration) have been thoroughly examined [6,24], there are few studies concerning the *AO* promoter sequence and the transcriptional regulation of this gene. Kisu *et al*. described a pumpkin *AO* promoter and analyzed the transient expression in pumpkin fruit tissues after fusing the promoter to the β-glucuronidase reporter gene [25].

The *GhAO1* promoter contains classical eukaryotic elements, such as a TATA-box and CAAT-box, for transcription initiation. In addition, some plant transcription factor binding sites were observed, including MYB and HSE (Fig 1). The significant roles of the transcription factor MYB and heat shock proteins in cotton fiber development have been explored [26–29], suggesting that MYB and heat stress play roles in the regulation of *GhAO1* expression. In addition, some light responsive elements, such as the G-box and I-box (Fig 1), were also identified, indicating that *GhAO1* might be a light modulated gene. Similar studies have demonstrated that *AO* expression is light dependent in tobacco [6, 30]. Moreover, root- and meristem-specific expression elements have also been identified, including root motifs and CAT-box sequences, respectively (Fig 1), implying that *GhAO1* might be involved in tissue and organ development. High AO activity has been detected in the root quiescent center (QC) and the stem cell niche [31].

*GUS* and *GFP* gene fusion is most commonly used as a reporter gene expression system to detect promoter activity, and 5’-deletion analysis is considered a useful method to determine the function of cis-elements of the promoter [32,33]. Promoter fusion constructs induce the effective and stable expression of the reporter gene GUS or GFP in transformed plants using transient expression methods [17–18]. In the present study, the *pGhAO1*::*GFP-GUS* fusion expression vector was constructed and transformed into tobacco leaves, demonstrating that the promoter of the *GhAO1* gene could significantly drive the expression of *GUS* and *GFP* based on GUS staining (Fig 2) and fluorescence detection (Fig 3) analyses, respectively. High GUS activity was observed in transformed tobacco leaves, whereas, less GUS activity was observed in transformed P-1040 tobacco leaves (Fig 4), suggesting that a silencer might be present and suppress promoter activity.

The promoters of several cotton genes highly expressed in fiber cells have been isolated, and the promoters of these genes, *GhLTP6*, *GhLTP3*, *GhRGP1*, and *GhGlcAT1*, have been examined in transgenic tobacco plants. The *GhAO1* promoter was cloned from cotton, and activity validation was realized using transformed tobacco plants. Auxin promotes cell elongation through the generation of ROS, affecting cell wall composition and microtubule assembly [6]. The *GhAO1* promoter contains an auxin responsive element at the -1,609 bp position (Fig 1), and a similar result has been reported for the pumpkin *AO* promoter, which contains a cis-acting region responsible for auxin regulation [25]. *GhAO1* expression was induced by IAA treatment (Fig 5), consistent with the result reported for pumpkin [9]. The oxidation in apoplasts that is catalyzed by AO might lead to an absence of auxin-dependent reactions, resulting in plants insensitivity to IAA. These results suggest a potential link between auxin and the transcriptional regulation of *AO* expression, which are associated via the auxin responsive element. The overexpression of the *AO* gene in tobacco induces the accumulation of H_2_O_2_, increases MAPK enzyme activity and decreases plasmalemma-localized two-pore Ca^2+^ channel-associated gene (*NtTPC1B*) expression [18]. The functions of H_2_O_2_ and calcium ion signals in fiber elongation have previously been described [34–37].

Promoter sequence analysis showed that a gap in the A genome/sub-genome might inhibit the expression of *GaAO* by deactivating the promoter regions of the *GaAO* gene. The 950-bp substitution in the promoter region in the A genome between -1502 to -1787 bp is adjacent to the auxin response element, as determined based on a GUS reporter assay (Fig 5), suggesting that this region is important for the activity of the *AO* promoter. Furthermore, a 16-kb insertion in the At sub-genome in Gh from -801 to -962 bp might block transcription factor binding, resulting in the loss-of-function of the *GaAO* promoter in upland cotton (Fig 6A). We observed slight differences in both CDS and promoter regions of *AO* gene between the At sub-genome sequence and that of other sequences. This may potentially reflect variations between the cotton varieties used for genome sequencing (TM-1) and promoter cloning (Xuzhou 142). However, the mechanisms by which these insertions abrogate the activity of *AO* promoter in the A genome/sub-genome were not determined in the present study. Our results suggest a potential mechanism in which the *GhAO1* promoter might be involved in cotton fiber cell growth via an auxin-mediated signaling pathway.

## Supporting Information

S1 Fig Analyses of *GhAO1* expression pattern and total AO activity during different cotton fiber elongation stages. Total RNA isolated from tissues of cotton ovules and fibers of various development stages were used for QRT-PCR analysis. The cotton ubiquitin gene, UBQ7 (Genbank accession no. AY189972) was used as the template control. Total AO enzyme activity was determined using samples prepared from the different growth stages indicated. The QRT-PCR and enzyme activity results were obtained from three independent experiments.
(JPG)
