# Supplementary figures and images for: Cloning and Functional Analysis of the Promoter of an Ascorbate Oxidase Gene from Gossypium hirsutum
Source: PLoS One. 2016 Sep 6;11(9):e0161695. doi: 10.1371/journal.pone.0161695 (PMC5012575; doi:10.1371/journal.pone.0161695)

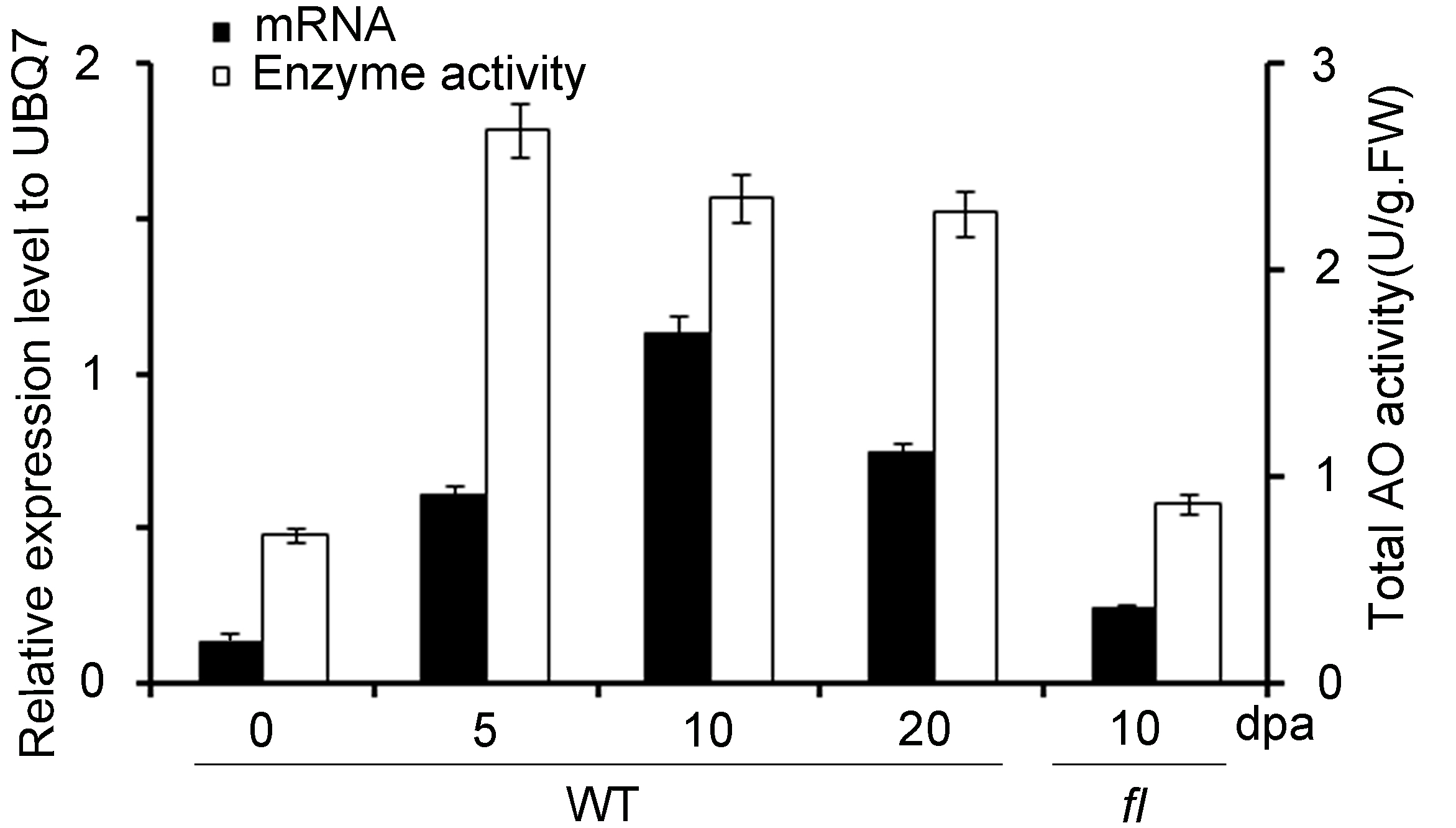

Supplement: S1 Fig — Total RNA isolated from tissues of cotton ovules and fibers of various development stages were used for QRT-PCR analysis. The cotton ubiquitin gene, UBQ7 (Genbank accession no. AY189972) was used as the template control. Total AO enzyme activity was determined using samples prepared from the different growth stages indicated. The QRT-PCR and enzyme activity results were obtained from three independent experiments. (JPG) [file pone.0161695.s001.jpg]
